# Flavonoids as Epigenetic Modulators for Prostate Cancer Prevention

**DOI:** 10.3390/nu12041010

**Published:** 2020-04-06

**Authors:** Simona Izzo, Valeria Naponelli, Saverio Bettuzzi

**Affiliations:** 1 Department of Medicine and Surgery, University of Parma, Via Volturno 39, 43125 Parma, Italy; simona.izzo@unipr.it (S.I.); saverio.bettuzzi@unipr.it (S.B.); 2 National Institute of Biostructure and Biosystems (INBB), Viale Medaglie d’Oro 305, 00136 Rome, Italy; 3 Centre for Molecular and Translational Oncology (COMT), University of Parma, Parco Area delle Scienze 11/a, 43124 Parma, Italy

**Keywords:** prostate cancer, flavonoids, epigenetic regulation, miRNAs, green tea catechins, natural compounds, lncRNAs, apoptosis, cell cycle arrest, chemoprevention

## Abstract

Prostate cancer (PCa) is a multifactorial disease with an unclear etiology. Due to its high prevalence, long latency, and slow progression, PCa is an ideal target for chemoprevention strategies. Many research studies have highlighted the positive effects of natural flavonoids on chronic diseases, including PCa. Different classes of dietary flavonoids exhibit anti-oxidative, anti-inflammatory, anti-mutagenic, anti-aging, cardioprotective, anti-viral/bacterial and anti-carcinogenic properties. We overviewed the most recent evidence of the antitumoral effects exerted by dietary flavonoids, with a special focus on their epigenetic action in PCa. Epigenetic alterations have been identified as key initiating events in several kinds of cancer. Many dietary flavonoids have been found to reverse DNA aberrations that promote neoplastic transformation, particularly for PCa. The epigenetic targets of the actions of flavonoids include oncogenes and tumor suppressor genes, indirectly controlled through the regulation of epigenetic enzymes such as DNA methyltransferase (DNMT), histone acetyltransferase (HAT), and histone deacetylase (HDAC). In addition, flavonoids were found capable of restoring miRNA and lncRNA expression that is altered during diseases. The optimization of the use of flavonoids as natural epigenetic modulators for chemoprevention and as a possible treatment of PCa and other kinds of cancers could represent a promising and valid strategy to inhibit carcinogenesis and fight cancer.

## 1. Introduction

Prostate cancer (PCa) is a multifactorial disease. Abnormal growth of cells may become invasive, leading to the spread and metastasizing to different tissues in the body. Worldwide, PCa is the second most frequently diagnosed cancer and the fifth leading cause of cancer death [1]. Due to the increase in testing, PCa diagnosis has grown exponentially in recent decades. Nowadays, this disease is considered the most common, life-threatening tumor affecting the European male population [1]. However, most prostate cancers are indolent: they rarely progress towards clinical significance. However, it is difficult to discriminate between clinically-significant and clinically-insignificant PCa [2]. When a man receives a PCa diagnosis, the most likely treatment options are surgical removal of the gland, chemotherapy, and/or radiotherapy. These are especially effective in the early stage of the disease but become useless for locally advanced or metastasized PCa. In fact, some patients eventually develop an aggressive form that resists treatment (castration-resistant prostate cancer, CRPC). When this happens, after an initial response, PCa cells no longer respond to androgen deprivation therapy [3,4,5]. The etiology of PCa has not be fully clarified; however, it is known that, in addition to genetic and biological factors, such as ethnicity, predisposition, and geographic location, environmental factors like diet and lifestyle can strongly influence the risk of PCa [6,7,8,9,10,11,12].

Cancer chemoprevention, whose definition is the use of non-toxic natural or synthetic molecules to prevent, inhibit, or reverse the onset and progression of cancer, is currently one of the most studied and promising fields of research [13,14]. Due to its high prevalence, long latency, and slow progression, PCa is an ideal target for chemoprevention strategies. Many research studies have highlighted the positive effects of natural compounds, such as vitamins, phenols, flavonoids, and mineral substances on chronic diseases [15,16,17,18]. In particular, polyphenols are one of the most studied class of phytochemicals because of their anti-inflammatory, antiviral, anti-allergic, antioxidant, and antitumoral effects [19,20,21]. The most common classes of polyphenols are flavonoids and phenolic acids, representing approximately 60% and 30% of all natural polyphenols, respectively [22,23].

## 2. Flavonoids

### 2.1. Structure and Metabolism

Flavonoids are a class of secondary plant metabolites generally used by vegetables for their growth and defense against microbes [24].

Flavonoids cannot be synthesized by humans and animals but are important components in the human diet. They are associated with many healthy effects due to their anti-oxidative, anti-inflammatory, anti-mutagenic, anti-aging, cardioprotective, anti-viral/bacterial, and anti-carcinogenic properties, together with their capacity to modulate enzyme function [24].

**Figure 1:**
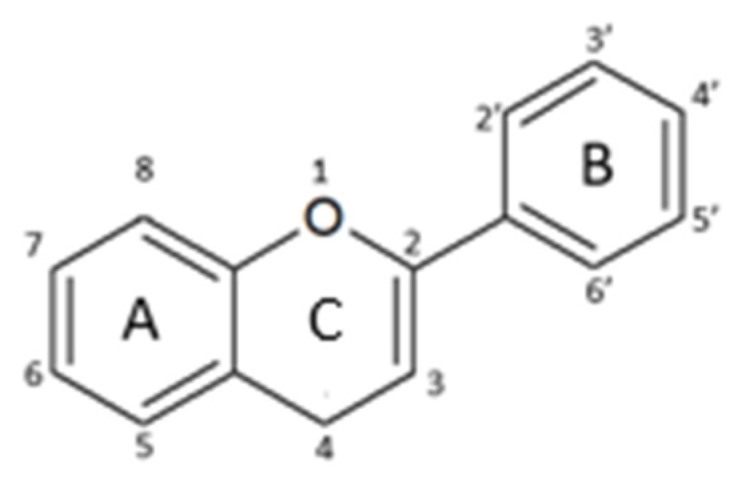
General structure of a flavonoid.

Their structure is based upon two benzene rings (A and B ring) that are linked via a heterocyclic pyran ring (C ring) containing oxygen (Figure 1) [25].

Based on their structural differences, flavonoids can be divided into (Table 1):

- Flavones: have a double bond between C2C3 and a C4-oxo function;
- Flavonols: are flavone analogues with a 3-hydroxylic group;
- Flavanones: are flavone analogues but with a C2-C3 single bond;
- Isoflavonoids: have the B ring attached at C3, rather than C2 position of the C ring;
- Flavanols or catechins: are the 3-hydroxy derivatives of flavanones, they have the hydroxyl group always bound to position 3 of the C ring;
- Anthocyanins: have a basic chemical structure with a flavylium cation, which binds the hydroxyl and/or methoxyl group(s) in R₁, R₂, and R₃ position.

Generally, the color and taste of food are conferred by flavonoids, which also play a role in the prevention of fat oxidation and the protection of vitamins and enzymes [25]. The scientific interest in these compounds has increased as of late, mainly due to their potential to promote health and reduce the risk of disease.

Flavonoids are generally found in plants as free aglycones (the basic form of flavonoids), glycosides, and methylated derivatives.

The physical and chemical properties of dietary flavonoids affect their ability to be absorbed by the intestine; while the small intestine can easily absorb flavonoid aglycones, the flavonoid glycosides, which are bound to sugars and represent the majority of these polyphenols, must be converted to the a glycan form by β-glucosidases before being absorbed [25,26]. No free flavonoids are present in plasma or urine since the absorbed flavonoids are immediately conjugated in the liver.

Many studies performed both in vitro and in vivo demonstrated the anti-tumoral effects of flavonoids on different types of cancer [27,28,29]. Epidemiological studies have shown an association between plant-derived flavonoids consumption and cancer risk reduction [29,30,31].

Regarding more specifically PCa, in vivo pre-clinical studies have evidenced the chemopreventive effect of natural compounds in PCa xenograft mouse models and in transgenic mouse models. Although the association between polyphenols intake and tumorigenesis of PCa needs to be confirmed by large-scale epidemiological data [32], since results are sometimes inconsistent and conflicting, there is a general agreement that consumption of flavonoids in the diet significantly reduces PCa risk [33,34,35,36,37].

The bioavailability of the different flavonoids differs between individuals. For this reason, there is a lack of correlation between the dose administered and the concentration measured in the human body. Moreover, metabolites, rather than pure polyphenol, often show the greatest biological activity [38,39]. When studying pharmacokinetics, each step, as well as all the enzymes implied in its absorption, modification, and transport, should be taken into account for a better understanding of the beneficial effects of flavonoids [40]. Flavonoids are generally well tolerated by prostate cells [41]. However, the potential toxic effects of excessive flavonoid intake are often ignored; it is likely that they act as mutagens, inhibitors of key regulatory enzymes, or as pro-oxidant molecules. Therefore, it is important to not exceed the intake levels that have been demonstrated useful in vivo [42]. No important side effects have been demonstrated when given up to 1 g/die in humans [43]. Many different kinds of polyphenols have been studied in the attempt to kill PCa cells [44,45,46,47,48,49,50,51].

### 2.2. Biological Activity of Flavonoids

Flavonoids, thanks to their anti-oxidant, anti-cancer, anti-microbic, anti-viral and anti-aging activities, cause a variety of biological effects on different types of cells.

#### 2.2.1. Anti-Oxidant and Pro-Oxidant Activity

Reactive oxygen species (ROS) play an important role in many human diseases, such as cancer or neurodegenerative diseases. In fact, ROS can combine with and oxidize many biomolecules, causing damage to cells, tissues, and organs [52,53]. Hydroxyl OH•, superoxide O_2_•−, nitric oxide NO•, nitrogen dioxide NO_2_•, peroxyl ROO•, and lipid peroxyl LOO• are only a few examples of the most common reactive species constantly formed during cell metabolism. They are believed to contribute to cellular aging, mutagenesis, and carcinogenesis [54,55,56].

The anti-oxidant effects of flavonoids are explained by the number and arrangement of the hydroxyl groups around the nuclear structure [25].

Anti-oxidant activity is exerted through two major modalities: flavonoids can act as radical scavengers that either prevent the cellular damage produced by ROS or prevent the generation of ROS in the first place.

Flavonoid action is mediated by inhibition of the enzymes involved in ROS generation (such as glutathione S transferase, mitochondrial succinoxidase, NADH oxidase), or by an increase of anti-oxidant and detoxifying enzymes levels (such as glutathione peroxidase, glutathione reductase, and catalase) [25,57]. Reduction of ROS mediated by flavonoids is demonstrated in different PCa cell lines [58,59].

Moreover, flavonoids exhibit a pro-oxidant action, which is mainly ascribable to their ability to strongly chelate metal ions, such as Cu and Fe. The generation of a radical or a redox complex with a transition metal ion can cause damage in the DNA chain and mutations in gene expression [60]. For example, quercetin has been described as acting as a pro-oxidant or an anti-oxidant molecule, depending on the PCa cell line; in DU-145 cells, quercetin administration led to a ROS increase, while in LNCaP and PC3 cells, a ROS quenching activity was measured [61].

#### 2.2.2. Anti-Proliferative and Cytotoxic Effect

Multiple signaling pathways that play an important role in cancer development are the targets of many flavonoid compounds. Many of the protein effectors, which are found in multiple pathways, are improperly activated or abnormally expressed in several cancers. One of the most studied pathways involves Ras/Raf/Mitogen-activated protein kinase kinase (MEK)/Mitogen-activated protein kinase (MAPK) signaling. The binding of a ligand, or a growth factor, to a tyrosine kinase (RTK) receptor activates Ras, which stimulates the activation of several protein kinases such as Raf. Raf phosphorylates MEK1/2 which activates MAPKs or MAP kinase kinase (MKK) signaling. The downstream effectors of the MAPK include extracellular signal–regulated protein kinases (ERKs) and 90-kDa ribosomal S6 kinases (RSKs), while MKK triggers c-Jun N-terminal kinases (JNKs). In cancer cells, including those of the prostate, many of these kinases are constitutively activated, thus causing cell transformation and tumor growth [62].

One of the main targets of this pathway is activator protein 1 (AP-1), which is involved in cell-cycle progression. The main targets of AP-1 belong to two subfamilies of transcription factors, Jun and Fos. Jun and Fos activate other transcription factors, such as activating transcription factor 2 (ATF 2), cAMP response element-binding, nuclear factor of activated T-cells, or Sma- and Mad-related protein (SMAD) protein. The result of activation of this pathway is the increase of proliferation, angiogenesis, metastasis, and survival. Flavonoid compounds, such as delphinidin, quercetin, and myricetin, have been shown to interact with MEK, inhibiting its activity in an ATP noncompetitive fashion [63,64,65].

Flavonoids, such as luteolin or myricetin, are also able to exert their antitumor effect through direct binding to Fyn or Src proteins, non-receptor tyrosine kinases that can activate the Ras/Raf/MEK/MAPK pathways in the ATP-binding site [66,67].

Phosphoinositide-3-kinase (PI3K)/phosphatase and tensin homolog (PTEN)/Akt/ mammalian target of rapamycin (mTOR) signaling represents another pathway that is dysregulated in different types of cancer and can be hit by flavonoid compounds. PI3K, Akt, and mTOR are oncogenic proteins, while PTEN is a tumor suppressor. The oncoprotein Akt can be activated by mutations in PI3K, by loss of expression or activity of PTEN, or in response to induction by growth factors. mTOR, an effector of Akt signaling, controls nuclear factor kappa-light-chain-enhancer of activated B cells (NF-kB) transcriptional activity that modulates cell proliferation and apoptosis [68]. Flavonoids act by means of the inhibition of phosphorylation and activity through their binding to Akt or PI3K, or they can act by reducing UVB-induced phosphorylation of Akt. The effects of the inhibition, in general competitive with ATP, of PI3K or Akt by flavonoids, include suppression of Cyclooxygenase-2 (COX-2) expression, and a decrease in cell migration and metastasis-inducing proteins, such as metalloproteinase (MMP)-9, MMP-13, and vascular endothelial growth factor (VEGF) [68,69,70]. These pathways are fully and clearly reviewed in by Bode and Dong [62], and also described in PCa cells [61,71,72,73].

Androgens and androgen receptors are required for the development of the male urogenital system and are involved in prostate cells proliferation. In fact, androgen deprivation is the first therapeutic approach for PCa, generally leading to a first positive response of patients, until they later become “androgen refractory”. Flavonoids exert anti-androgenic effects through multiple mechanisms, such as inhibition of transactivators of androgen receptors, inhibition of the androgen receptor activity, or even by direct competition, mainly due to the structural similarity between these natural compounds and natural hormones [57].

#### 2.2.3. Cell Cycle Arrest and Apoptosis Induction

Flavonoids have been shown to decrease cell viability of cancer cells through induction of cell cycle arrest and activation of apoptosis. Cyclins and cyclin-dependent kinases (CDKs) form the main regulator complexes of the progression through the four phases of the cell cycle. In normal cells, the cell cycle is tightly regulated. In contrast, the cell cycle control is usually lost in tumor cells. Most flavonoid compounds induce cell cycle arrest through the alteration of cyclin levels: apigenin was found to reduce the level of cyclin D1, causing cell cycle arrest in the G0/G1 phase for PC3 and LNCaP PCa cell lines, and in G2/M phase for DU-145 cell line [74,75]. Polyphenon E, a standardized catechin extract, caused a G0/G1 cell cycle arrest in PNT1 cell line, and a G2/M arrest in PC3 cells [76]. Quercetin administration has been associated with a reduction in the levels of cyclin E, cyclin D, and CDK2 in PC3 cells, with a consequent arrest of cell cycle in G0/G1 phase [57,77].

Apoptosis is a form of programmed cell death caused by a set of precise cellular events that change the morphology and the functions of the cell. Apoptosis is important for tissue integrity and development; it can be initiated by the activation of cell death receptors via caspases-8 and -10 activation (extrinsic pathway) or by a cell stress sensed and triggered by activated mitochondria via activation of executioner caspases (intrinsic pathway). In normal cells, apoptosis is fundamental for the maintenance of tissue homeostasis and for the destruction of abnormal cells, such as tumor cells, which, conversely, have developed mechanisms to circumvent this process, such as the reduction of caspases expression or the loss of activity of cell death receptors.

Flavonoids have been shown to induce apoptotic death in many cancer cell lines [78,79].

In PCa cells, for example, apigenin has been shown to induce death receptor 5 and pro-apoptotic factors like tumor necrosis factor (TNF)-related apoptosis-inducing ligand (TRAIL), or higher levels of caspase-3 and -8 in cancer stem cells [57,73,80]. Fisetin decreased the activity of NF-kB in DU145 and PC3 cells and caused the activation of caspase-3 and -8 concomitantly with the disruption of the mitochondrial membrane [81]. Quercetin, in combination with EGCG, induced p53-mediated apoptosis in LNCaP and PC3 cells [82]. Polyphenon E induced endoplasmic reticulum stress, leading to death by anoikis in PNT1a and by necroptosis in PC3 cells [76].

## 3. Epigenetic Modifications Induced by Flavonoids

### 3.1. Epigenetics in Prostate Cancer

The term “epigenetic modifications” describes a stably hereditable change in DNA or associated proteins that affect and alter gene expression without modifying the DNA sequence [83]. These changes alter the structure of the chromatin and have an impact on the accessibility of promoters to the transcriptional machinery [84]. Possible alteration of gene expression may cause aberrant induction, or inhibition, of the main intracellular enzymatic and molecular cascades, leading to many diseases, including cancer. During the transformation of prostate cells into cancer cells, epigenetic alteration was frequently observed, and in most cases, it was found to precede any genetic mutations [85]. The main epigenetic modifications include DNA methylation, histone modification, nucleosome positioning, and expression of non-coding RNAs [83].

#### 3.1.1. DNA Methylation

DNA methylation consists of the covalent addition of a –CH_3_ group to the cytosine ring of the CpG dinucleotide. This dinucleotide is generally present at high density in specific, short regions of the human DNA (CpG islands). When these CpG islands are unmethylated, the chromatin is accessible, and the transcriptional machinery can easily attach the DNA and proceed towards gene expression. In all normal tissues, promoter regions are generally present in CpG islands in non-methylated form, while in PCa cells the promoters are commonly hypermethylated; consequently, genes are repressed. In cancer cells, the CpG islands present in suppressor genes are often hypermethylated, resulting in gene inactivation. This would sustain cells growth and spreading of cancer cells [86]. In PCa, methylation of hormone receptor genes, such as androgen, estrogen, or progesterone receptors, is a late stage event of carcinogenesis [87]. Genes involved in the regulation of important cellular processes, such as DNA repair, migration, cell cycle regulation, and apoptosis have been shown to have altered behavior due to aberrant promoter, or gene methylation, in PCa cells [88,89,90,91,92,93].

DNA methyltransferases (DNMTs) are the enzymes responsible for DNA methylation. They include three isoenzymes: DNMT1, which is involved in maintaining the post-replicative methylation pattern of DNA, and DNMT3a and DNMT3b, which are recruited for de novo DNA methylation [86,94]. Late stage PCa tissues have methylated androgen receptor promoters [86].

Another epigenetic change commonly found in human cancers is the loss of DNA methylation that is frequently associated with increased transcription. In PCa, DNA hypomethylation is generally observed in the late stage of cancer progression, when lymph nodes have been invaded [86,95].

#### 3.1.2. Histone Modifications

Histones are positively charged proteins with the role of packing DNA in ordered structures called nucleosomes. Nucleosomes are the basic repeating structural units of chromatin. Histone covalent modifications, such as acetylation, phosphorylation, and methylation, determine chromatin structure changes that influence gene expression. If the chromatin is open (euchromatin), transcription is activated, whereas, when the chromatin is closed (heterochromatin), transcription is inhibited.

Histone acetylation is the covalent addition of an acetyl group to the lysine residues present in the histone tail by the histone acetyltransferases (HATs); this precise modification allows the expression of genes. Therefore, HATs are considered transcriptional co-activators. Conversely, histone deacetylases (HDACs) are a family of enzymes acting as co-repressors of transcription, as they de-acetylate the histone tails of the nucleosome, thus favoring the gene repression [96].

Generally, an increased activity of HDACs is correlated with increased prostate-specific antigen (PSA) serum levels and tumor cell invasiveness in PCa cells.

Another epigenetic modification is catalyzed by histone methyltransferases (HMTs), enzymes able to transfer methyl groups from S-adenosyl methionine (SAM) to a lysine or an arginine residue of the histone tail, while the same groups are removed by histone demethylases (HDMs). The activation or repression of genes transcription depends on the position and the level of methylation of the histones. Prostate cancer-specific transcription profiles are often related to the regulation of histone methylation [97,98]. Histone phosphorylation is mediated by several kinases (stress-activated protein kinase, kinase Aurora B, etc.), and is generally related to the cell cycle. The phosphorylation of serine, located four residues from the C terminus of H2A histone family member X (H2AX), which is a marker of DNA double strand break, an alteration leading to genomic instability and, eventually, to cancer [99]. Phosphorylation of histone H2AX, which is catalyzed by kinases belonging to the family of phosphatidylinositol 3-kinase-related protein kinases, determines the chromatin relaxation. In PCa, the presence of phosphorylated histone H2AX is linked to prostate cell transformation [100].

#### 3.1.3. miRNA

MicroRNAs (miRNAs) are small non-coding RNAs composed of 18–25 nucleotides that specifically regulate gene expression. They exert oncogenic (oncomirs) or tumor suppressor effects, often interacting with the 3’ region and, rarely, with the 5’ untranslated region of the mRNA target. In this way, miRNA affect the stability of the transcripts and/or stop gene translation. One miRNA can interact and modulate the expression of several genes; in consideration of this fact, few miRNAs are enough to cause an amplified dysregulation of a broad range of important cellular processes. Moreover, several miRNAs can target the expression of a specific mRNA [101]. The alteration of miRNA expression can be due to genetic alterations, promoter hypermethylation, or epigenetic modifications and can promote and coordinate cancer onset and progression.

Several studies describe an alteration of the miRNA profile in PCa whereby miRNAs are used as biomarkers to distinguish indolent and aggressive cancers. The most common oncomirs found in PCa are miR-21, miR-32, miR-221, miR-222, miR-181, miR-18a, and miR-429. miR-21 and miR-32 are two androgen-regulated oncogenic miRNAs that target tumor suppressors, thus promoting cell proliferation. In particular, miR-21 targets reversion inducing cysteine rich protein with Kazal motifs (RECK) gene and promotes cell invasion and metastasis through the control of matrix metalloproteinase 9 (MMP-9) or interacts and downregulates phosphatase and tensin homolog (PTEN) and programmed cell death (PDCD4), favoring apoptosis inhibition and tumor progression. miR-32 targets B-cell translocation gene 2 (BTG-2) and phosphoinositide-3-kinase interacting protein 1 (PI3KIP1) regulating cell proliferation, survival, and cell invasiveness. miR-221, miR-222, and miR-429 modulate the expression of cell cycle regulators. miR-18a acts as an oncomir: it targets the serine/threonine kinase 4 (STK4) 3’ untranslated region, inducing its downregulation and promoting tumor survival [101,102].

The tumor suppressor miRNAs are generally downregulated in cancer cells, including PCa cells, and promote proliferation, metastasis, and cell invasiveness. In PCa miR-143, miR-145, and members of the miR-200 family play a major role in cell migration and tumorigenesis due to the fact that their downregulation activates epithelial–mesenchymal transition (EMT) [103,104]. EMT is a process through which epithelial cells assume the features of mesenchymal stem cells, able to differentiate in a broad range of cells, thus acquiring invasive and migratory properties. This effect seems to be mediated by the activation of the translation factors zinc-finger E-box binding homeobox 1 and 2 (ZEB1 and ZEB2) [105]. miR-29b, miR-205, miR-940, and miR-218 downregulation in PCa is related to an increase in cell invasiveness and migration, together with an increase in mesenchymal phenotype of prostate cells [101,103,106].

Other miRNAs, such as miR-15a and miR-16, target important oncogenes, such as Bcl2, Mcl1, CCnd1, and Wnt3, all involved in apoptosis induction. In PCa, these miRNAs are generally downregulated, favoring the inhibition of apoptosis and tumor progression. The pleiotropic effects exerted by tumor suppressor miRNAs include the modulation of androgen receptor (AR) genes expression (miR-488 *, miR-125b, miR-155a, miR-27a) and cell proliferation stimulation (miR-497, miR-296-5p). Loss of miR-101 in PCa cells modifies histone methylation and, consequently, gene expression [101]. Another important miRNA in PCa cells is miR-195. Its abnormal expression has been related to poor survival of PCa. MiR-195 has been shown to bind to, and target, the 3’ untranslated region of the clusterin (CLU) gene, thus regulating docetaxel resistance of PCa cells [107]. CLU is a secreted glycoprotein found to be involved in neurodegeneration, aging, and cancer. The specific role of CLU in tumorigenesis is still a matter of debate, as its expression has been found altered (i.e., upregulated or downregulated) in different kinds of cancer. Downregulation of CLU in naïve cancers is, by far, the prevailing condition that is found. CLU may be tumor-suppressive at the initial stages of carcinogenesis and tumor-permissive at late stages or in therapy-resistant cancers [108]. CLU is downregulated in human PCa progression and during the development of PCa in the transgenic adenocarcinoma of the mouse prostate (TRAMP) model [109]. Its expression is restored in TRAMP mice responding to chemoprevention with green tea catechins [41]. Knocking down CLU in the TRAMP model generated a more aggressive kind of PCa [110]. The silencing of CLU expression in the TRAMP model promoted activation of NF-κB and transcriptional upregulation and increased activity of MMP-2 and MMP-9 [111].

The let-7 family members of miRNAs function as tumor suppressors: let-7a and let-7c are generally downregulated in PCa and positively impact the cell proliferation rate [101].

#### 3.1.4. Long Noncoding RNA (lncRNA)

Recently, several studies have focused on the importance of noncoding RNAs on genome dynamic expression. The protein-coding RNAs constitute less than 2% of the human genome, meaning that the majority of the human transcriptome produces RNAs that are not translated into proteins. lncRNAs have a length of more than 200 nucleotides, most are polyadenylated, transcribed by RNA polymerase II, and able to bind DNA, RNA, and proteins. More targeted research is being done on lncRNAs due to the fact that different regulatory roles of these molecules have been demonstrated. Epigenetic modulation is the most common method of lncRNAs regulation of gene expression, and it generally results in transcriptional repression. LncRNAs cooperate with polycomb repressive complexes (PRC) to control the transcriptional machinery activity. Other mechanisms by which lncRNAs modulate gene expression involve mRNA processing through alteration of transcript stability, processing and translation, interaction with miRNAs, gene enhancers and repressors, or transcription factors affecting transcript production and transport. In this way, lncRNAs can act as oncogenes or tumor suppressors, thus influencing important cellular processes. Many lncRNAs are over-, or under-expressed in PCa, where they have been found to interact with and modulate AR signaling or to affect fundamental cell pathways. The molecular mechanisms of lncRNAs in PCa progression and their potential role as biomarkers or therapeutic targets need to be explored more deeply [112,113]. Nevertheless, flavonoids have been found as lncRNA modulators in different cancer cell types [114].

## 4. Flavonoids as Epigenetic Modulators in Prostate Cancer

The pleiotropic effects exerted by flavonoids in PCa cells can also be explained by epigenetic changes in gene expression and chromatin organization. Epigenetic alterations have been identified as key initiating events frequently occurring in several cancers. In this part of the review, we will focus on the ability of flavonoids to restore the “normal epigenetic marks” that are often altered in tumor cells. This feature is the reason why epigenetic therapy as a promising treatment option for multiple cancer types.

### 4.1. Flavones

Apigenin is a natural flavone (4’,5,7-trihydroxyflavone) that is present in high quantities in grapefruits, onions, parsley, and chamomile [115]. The growth inhibitory effects found in cancer cells after apigenin administration have been attributed to multiple mechanisms of action. Apigenin affects cell cycle regulation, apoptosis, immune response stimulation, and cell migration. Many pathways have been found to be modulated by this flavone, including PI3K/protein kinase B (Akt) MAPK/ERK, Janus kinases (JAKs), signal transducer and activator of transcription proteins (STATs) (JAK/STAT), NF-κB, and Wnt/β-catenin [116].

**Figure 2:**
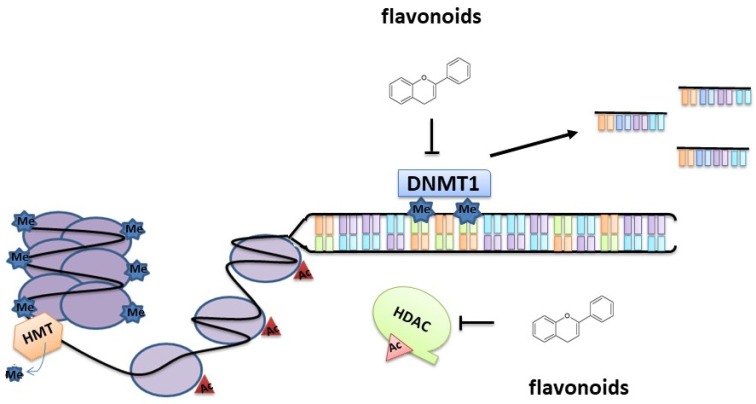
Epigenetic mechanisms of flavonoid action. Flavonoids (apigenin, silibinin, catechins) can act as histone deacetylase (HDAC) inhibitors by promoting the transcriptional expression of genes involved in cell cycle arrest and apoptosis induction. Flavonoids (genistein, catechins) promote the downregulation of DNA methyltransferase (DNMT)1 that leads to the demethylation, and consequent reactivation, of methylation-silenced genes.

PCa, PC3, and 22Rv1 cells, exposed to 20 and 40 µM of apigenin for 24 h, showed a decrease of HDAC activity comparable to that obtained using the well-known HDAC inhibitor trichostatin (TSA). Specifically, apigenin caused the downregulation of HDAC1 and HDAC3, both at the protein and mRNA levels, with a concomitant increase of the acetylation of H3 and H4. As a consequence, this enhanced the accessibility of DNA promoters to transcription factors, increasing the synthesis of the cell cycle regulator protein p21/waf1 in PCa cells [50]. p21/waf1 controls cell cycle progression primarily through inhibition of cyclin-dependent kinase 2 (CDK-2) (Figure 2).

It is a common target for HDAC inhibitors (HDACi). PCa cells showed cell cycle arrest and apoptotic pathway induction after 24 h of apigenin administration [50]. In vivo studies conducted on PC3 xenografts in athymic nude mice confirmed the antitumoral action of apigenin. An oral intake of 20 and 50 mg/mouse/d over a period of eight weeks caused a marked decrease of HDAC activity, and HDAC1 and HDAC3 protein expression also decreased as well as a reduction in tumor growth. In apigenin-fed mice, p21/waf1 expression was higher than in control mice, and bax/bcl2 ratio shifted towards the induction of apoptosis [50]. In vitro and in vivo experiments demonstrated the pleiotropic effects of apigenin in different PCa models: a reduction of HDACs, ROS production enhancement, attenuation of NF-kB pathway, and caspase activation are only a few examples of the action mediated by this flavone, whose action is selective only towards cancer cells and does not affect normal cells [117]. The efficacy of apigenin as a single agent improves when administered in a combinatorial therapeutic approach with other chemotherapeutic agents or with other HDAC inhibitors. Recent results showed that apigenin synergistically potentiates tumor necrosis factor-related apoptosis by inducing ligand (TRAIL)-induced apoptosis in DU145 cells [117].

Luteolin (3′,4′,5′,7′-tetrahydroxyflavone) belongs to the flavonoid group. Its structure is very similar to quercetin, although, compared to quercetin, it is a less efficient radical scavenger. Luteolin has two benzene rings and a third ring containing oxygen. It is found in onion, broccoli, carrots, peppers, and apple skin [118] and exhibits a wide range of beneficial properties, including anticancer activity [119,120,121]. Luteolin modulates various signaling pathways involved in carcinogenesis [122,123].

PC3 and LNCaP cells treated with 10–60 μM of luteolin for 24, 48, and 72 h showed a marked dose-dependent reduction of cell proliferation and induction of apoptosis. The Authors noticed that 16 kinds of miRNA were down or upregulated in PCa cells exposed to luteolin. Specifically, the Authors focused on miR-301, which was found to be the most downregulated oncogenic miRNA after luteolin administration. The pro-apoptotic factor death effector domain containing 2 (DEDD2), a putative important mediator for death receptors, was indicated as a target of miR-301. The mRNA and protein levels of DEDD2 were found to have increased more than two times with miR-301 in both PCa cells [121].

Sakurai et al. described the co-administration of luteolin and/or gefitinib to human PCa PC3 cells as having had a greater effect on cell viability than the administration of either compound alone. The treatment was associated with a significant decrease of the expression of cyclin G-associated kinase (GAK), which has an important role in clathrin-mediated membrane trafficking and is often overexpressed in cancer cells. The downregulation of GAK is mediated by the upregulation of miR-630, which is associated to hyper-phosphorylation of tyrosine residues in epidermal growth factor receptor (EGFR) and alters the downstream signaling [124].

When luteolin binds to type II ^3^H-estradiol binding sites, identified as histone H4, the proliferation of normal and tumor prostate cells is inhibited in vitro and in vivo. Luteolin blocks the acetylation of histone H4 and regulates the expression of c-FOS, p21, and other genes playing key roles in the epidermal growth factor receptor signaling pathway and in cell cycle regulation [125].

Morin (3,5,7,20,40-pentahydroxyflavone) is a flavonoid isolated from plants belonging to the Moraceae family. Its structure is constituted by two aromatic rings linked by an oxygen-containing heterocycle. Morin administration induces apoptosis and was found to have anti-tumoral effects in several cancer cell lines [126,127,128].

In LNCaP (human PCa cells), morin at a concentration of 50 and 75 μM induced apoptosis and cell proliferation arrest after 24 and 48 h, respectively [129,130].

In a recent report, morin was shown to dysregulate the expression of several miRNAs, including miR-143, miR-146b, and miR-155 in PC3 and DU145 cells.

Specifically, the most effective result was the suppression of miR-155 after cell treatment with 50 μM of morin. The treatment of morin and/or paclitaxel led to the recovery of the expression of GATA binding protein 3 (GATA3) through inhibition of miR-155 [131]. In normal prostate, GATA3 is highly expressed, and it is involved in the regulation of prostate-specific antigen (PSA) genes, whereas, during cancer progression, its expression is gradually reduced. The treatment of PCa cells with morin caused an increase of GATA3 expression and a decrease of zinc finger E-box-binding homeobox 2 (ZEB2), transforming growth factor beta 1 (TGFB1), and murine double minute 2 (MDM2) levels. The chemo-sensitivity of PCa cells to paclitaxal is increased by morin addition, resulting in restoring the miR-155-suppressed expression of GATA3 [131].

Tricin (4′,5,7-Trihydroxy-3′, 5′-dimethoxyflavone) is a flavonoid with important beneficial effects for human health. It exhibited anti-oxidant activities, a protective action for liver function, and was effective against the flu virus [132]. A recent study reported the synergistic anti-proliferative effect of docetaxel (0.01 nM) and tricin (60 μM) on human PCa PC3 cells. Although the mechanism of action of this natural compound requires more investigation, the Authors found a significant decrease in miR-21 expression 48 h after the treatment of PC3 cells with 120 and 140 μM of Tricin. Mir-21 is generally overexpressed in men with metastatic PCa or resistant to docetaxel [133].

### 4.2. Flavonols

Quercetin is categorized as a flavonol. It has a hydroxyl group in 3,5,7,3’ and 4’ position. It is found in a variety of foods, such as apples, berries, onions, grapes, capers, tea, and tomatoes [134].

Quercetin has different intracellular molecular targets and affects multiple signaling processes that are altered in cancer cells by exerting anti-inflammatory, anti-oxidant, and anti-microbic effects.

Quercetin has been shown to slow down carcinogenesis through inhibition of a pro-proliferative signaling pathway, both in animal models and in human cancer cell lines [135,136,137].

Several studies have demonstrated that quercetin modulates the expression and the activity of several epigenetic enzymes, such as DNMTs, HATs, and nuclear HDACs [138]. The treatment of quercetin has also been found to reduce dose-dependent activity of some histone phosphorylases, such as aurora kinase (AURKA) A, B, and C, which are involved in the progression of the cell cycle [138].

A recent study by Yang et al. showed that the combination of quercetin and hyperoside (quercetin-3-O-galactoside), a flavonoid compound extracted from *Hypericum perforatum*, has a strong anticancer effect on PCa cells [139]. A possible mechanism responsible for growth arrest and the inhibition of metastatic spread involves the regulation of miR-21 expression in PC3 cells. CLU, whose expression is downregulated in PCa cells, has been identified as one of the targets of miR-21 in head and neck squamous cell carcinoma [140]. Quercetin and hyperoside administration lead to a decrease of oncomir miR-21 expression, with a resulting increase in the translation of target proteins like PDCD4. PDCD4 inhibits growth promotion through the suppression of the transactivation of the AP-1 promoter via c-Jun [141] and through the inhibition of the eukaryotic initiation factor 4A activity [139,142].

### 4.3. Flavanones

Silibinin is a natural flavonolignan. It is the most active compound of silymarin, a standardized extract from the seeds of milk thistle (Silybum marianum). Silibinin is composed of silybin A and silybin B, two diastereoisomers of almost equimolar concentration. Along with the stereoisomers dihydrosilybin, isosilybin, silychristin, and silydianin, silibinin represents the most abundant component of silymarin [143]. Silibinin affects multiple signaling and regulatory mechanisms and exhibits anti-tumor efficacy in many cancer types in vitro and in vivo. In PCa, it has been shown to inhibit tumor progression affecting cell proliferation, epithelial–mesenchymal transition, invasion, metastasis, angiogenesis, and apoptosis [143]. In a recent study, silibinin administration at concentrations of 25–75 µg/mL for 48 h was shown to reduce the expression levels of enhancer of zeste homolog 2 (EZH2), embryonic ectoderm development (EED), and suppressor of zeste homolog 12 (SUZ12), in DU145 and PC3 PCa cells. These proteins are components of the polycomb repressive complex 2 (PRC2), which has histone methyltransferase activity and catalyzes the methylation (adding one, two or three methyl groups) of histone 3 at lysine 27 (H3K27me1, 2, and 3). H3K27me3 is recognized as a hallmark of heterochromatin, which targets repressed genes. Silibinin treatment of PCa cells led to a strong decrease of EZH2 expression with a concomitant increase of the H3K27me3 levels; this effect can be explained by the modulation of the levels of Akt and its activated phosphorylated form (pAkt). pAkt phosphorylates the ser21 of EZH2, thus reducing the affinity of EZH2 for histone H3 with a consequent drop of H3K27me3. EZH2 is generally overexpressed in metastatic PCa cells and in patients with poor prognosis [144,145]. EZH2 protein controls other mechanisms of gene transcriptional repression, such as DNMT binding and HDAC recruitment. Furthermore, the effect of silibinin results in a modest increase of DNMT activity and a decrease in HDAC1 and HDAC2 expression levels, both actions leading to inhibition of gene expression [144].

### 4.4. Isoflavonoids

Genistein is the most biologically active isoflavone present in soybeans. It has been found to have in vivo and in vitro anticancer effects and anti-proliferative effects on several types of human cancers, including PCa [146,147].

Genistein has a human 17β-estradiol-like structure, and for this reason, it binds to the estrogen receptor, which is highly expressed in prostate epithelial cells, causing estrogenic and/or antiestrogenic effects [148]. The mechanisms through which genistein inhibits PCa progression involve different signaling pathways [149,150,151]. Genistein-dependent demethylating activity in cancer has recently been studied. Mahmoud et al. clearly showed that physiological doses of genistein (0.5–10 μM) significantly reduced the methylation levels of estrogen receptor (ER)-β promoter, resulting in increases in ER-β expression and induction of ER-β transcriptional activity in human PCa LNCaP and LAPC-4 cells. In PC3 cells, the effect was most likely not the same due to the low basal level of ER-β promoter methylation. Data reported by the research group indicated that a decline in DNMT1 and DNMT3b levels, without a change in DNMT3a levels, has been evidenced after genistein treatment. These enzymes, which are responsible for methyl group transfer to cytosine residues, were downregulated after 48 h of genistein administration and reverted tumor suppressor gene ER-β hypermethylation in PCa [152] (Figure 1).

In another study, a DNA microarray analysis was used to analyze the expression profile of genistein-treated PC3 and DU145 cells [153] versus controls. Results showed that genistein inhibited PCa cell growth through upregulation of tumor suppressor miR-34a, which directly targets HOX Transcript Antisense RNA (HOTAIR), a lncRNA that regulates key pathways in PCa invasion and metastasis [153]. A few recent studies focused on the interaction between miRNAs and lncRNAs in human cancer cells [154,155,156,157]. HOTAIR can function as a scaffold through binding and directing EZH2 and Lysine (K)-specific demethylase 1A (LSD1) to occupy the same genomic regions. It can also accelerate proteolysis through the facilitation of E3 ubiquitin ligases binding to their substrates. HOTAIR was also found to be an androgen-repressed lncRNA that can directly bind to AR to protect it from degradation in PCa. In castration-resistant PCa cells, where AR and androgen-repressed genes are upregulated, HOTAIR is present at higher expression levels in comparison to normal cells [158].

### 4.5. Catechins

Catechins are natural polyphenolic compounds belonging to the flavonoid family that are abundantly found in a variety of vegetables and fruits as well as in plant-based beverages. Green tea is the main dietary source of catechins [159]. Catechins, which account for more than 10% of the green tea leaves’ weight, are considered the compounds to which most of the beneficial effects of green tea can be ascribed [15,35,160]. Epigallocatechin-gallate (EGCG) is the most abundant (at least 50% of the total catechin content) and the most biologically active catechin present in green tea extracts. Many clinical studies have demonstrated the anti-proliferative, anti-oxidant, and anti-cancer effect of EGCG and, more generally, of a green tea catechins mixture [161], given pure or in combination with other natural compounds [162,163]. Although the anti-tumoral effects of EGCG have clearly been recognized, the molecular mechanism of action of catechins needs more investigation. As with many other natural compounds, catechins can also modulate epigenetic changes in gene expression and chromatin remodeling, as affects mainly DNA methylation and histone acetylation status [164,165,166,167,168]. EGCG has been reported to act on DNMTs, HDACs, and HATs expression levels and activity in different cancer cells, including PCa [169].

EGCG, at a concentration of 20 μM for 48 h, has been shown to competitively inhibit DNMT1 activity, causing demethylation of CpG islands and reactivation of methylation-silenced genes in human PCa PC3 cells [165]. Moreover, EGCG has been demonstrated to suppress androgen-dependent PCa cells growth and proliferation. Regulation of prostate cell proliferation and apoptosis appears to be modulated by androgen receptor (AR) acetylation [170]. AR, a hormone nuclear receptor that mediates androgen action in prostate cells, is regulated by HATs acetylation [170]. EGCG inhibited acetylation-dependent AR translocation to the nucleus in LNCaP cells [171]. Similarly, EGCG-treated mice bearing a 22Rv1 PCa cell xenograft showed a decrease in AR protein expression. EGCG was found to interfere with AR stability decreasing interdomain interactions, causing the inhibition of the transactivation functions and decreasing the expression of AR. These alterations were concomitant with an inhibition of miR-21 and an upregulation of miR-330, which acts as a tumor suppressor that is able to induce apoptosis of PCa cells in EGCG-treated mice in comparison to untreated controls [172].

In many studies, green tea catechins are administered in vivo or in vitro in the form of a standardized green catechins extract, called Polyphenon E, composed of EGCG (65%), EGC (4%), epicatechin (9%), epicatechin-3-gallate (6%), gallocatechin-3-gallate (4%), catechin-3-gallate (0.2%), gallocatechin (0.2%), catechins (1.1%), and caffeine (0.7%). A concentration of 10 μg/mL of Polyphenon E given to cell cultures roughly corresponds to 14.0 μM of EGCG.

Polyphenon E administered to LNCaP and PC3 cells for 24 h at a concentration of 10–80 μg/mL resulted in class I HDAC inhibition: the decrease in HDAC activity leads to hyperacetylation of histone H3 on the p21/waf1 and Bax promoters. These events are associated with cell cycle arrest and apoptotic death induction [173].

Exposure of human PCa LNCaP cells to 1–10 μg/mL of Polyphenon E for 1 to 7 days provoked the downregulation of DNMT1 mRNA and protein expression, which caused a reversal in glutathione-S-transferase P1 (GSTP1) CpG island hypermethylation and restoration of GSTP1 expression [174].

In a recent paper by Deb et al., green tea polyphenols were shown to inhibit the migration and invasion of PCa cells by reactivation of epigenetically silenced tissue inhibitor of metalloproteinase 3 (TIMP-3) and subsequent inhibition of metalloproteinases (MMPs) activity. MMPs, such as MMP-2 and MMP-9, are produced as inactive forms (pro-MMP) that need to be activated by cleavage to promote tumor cell migration. MMPs and TIMPs co-operate and regulate tumor progression. Recent studies suggest that epigenetic mechanisms regulate MMP-2 and MMP-9 activation, as well as genes involved in TIMPs control [175,176,177,178]. Human PCa DUPRO and LNCaP cells treated for 72 h with 10 μg/mL Polyphenon E or 20 μM EGCG showed an induction of TIMP-3 mRNA and protein. TIMP-3 expression was associated with downregulation of H3K27me3 (repressive histone mark) at the TIMP-3 promoter and an increase of H3K9/18 acetylation (transcription active histone signature) in human PCa cells. In green tea catechins treated cells, the Authors demonstrated a decrease in EZH2 and HDAC protein levels. Similar results have been described in tissue specimens obtained from PCa patients supplemented with Polyphenon E. In this clinical trial, patients received a total of 1.3 g of Polyphenon E during a six-week interval between prostate biopsy and radical prostatectomy [178].

### 4.6. Anthocyanidins

Delphinidin is one of the main anthocyanidins, characterized by a diphenylpropane skeleton. It is abundantly found in pigmented fruits and vegetables, such as pomegranates, berries, grapes, beets, and eggplants [179,180]. It exhibits anti-oxidant, anti-inflammatory, anti-angiogenic, and anti-cancer properties in different types of tumor cells [18,44]. In human PCa LNCaP cells, delphinidin administration (100 μM for 24 h) was found to increase the expression of active caspases. The active caspases led to HDAC3 cleavage, and HDAC3 inactivation resulted in p53 acetylation, activation, and oligomerization. The p53 protein activates important pro-apoptotic genes and plays a crucial role in the maintenance of genomic stability, thus inhibiting the growth of cancer cells. HDAC3 cleavage results in the p53-dependent induction of the expression of the pro-apoptotic proteins p21 and Bax [181].

Results are summarized in Table 2.

## 5. Conclusion and Perspectives

In this review, we overviewed the most recent evidence of the antitumoral effects exerted by dietary flavonoids, with a special focus on their ability to employ epigenetic regulation by modulating epigenetic enzyme activities in PCa. Epigenetic alterations were identified as key initiating events in several kinds of cancer. For this reason, epigenetic therapy is currently considered a valid strategy to inhibit carcinogenesis. Natural compounds are a pool of active molecules with a wide range of biological effects, including the epigenetic modulation of gene expression, which deserves further investigation. Many dietary flavonoids were found to reverse DNA aberrations that promote neoplastic transformation, particularly in the case of PCa. The epigenetic targets of flavonoid action include oncogenes and tumor suppressor genes, which are indirectly controlled through the regulation of epigenetic enzymes such as DNMTs, HATs, and HDACs. In particular, many flavonoids, such as apigenin, silibinin, and catechins, were found to be active at downregulating HDACs expression, mostly HDAC 1 and HDAC3. Downregulation occurs at both mRNA and protein levels, thus promoting the transcriptional expression of genes involved in cell cycle arrest and apoptosis induction. Other flavonoids, such as genistein or catechins, promote the downregulation of DNMT1 that leads to demethylation, and consequent reactivation, of methylation-silenced genes. In addition, flavonoids were found capable of interacting with miRNAs and lncRNAs. These are very important RNAs that modulate the expression of several genes and may cause the dysregulation of important cellular processes when their expression is altered during diseases. In particular, miR-21 is one of the most studied oncomirs, and represents a target for many flavonoids, including tricin, quercetin, and catechins. The downregulation of miR-21 expression promoted by flavonoids administration promotes cell cycle inhibition and the induction of apoptotic death of tumor cells; the pleiotropic effects of flavonoids may be explained by the regulation of the cascade triggered by miRNAs and lncRNAs.

Another promising issue is the combination of natural flavonoids with chemotherapeutic agents to potentiate the effect of pharmacological agents. This approach may have the potential to provide novel and more efficient strategies to fight cancer. To this end, it is necessary to better understand the epigenetic mechanisms and the players involved in the onset and progression of cancer by performing more clinical trials with flavonoids, compounds which are generally well tolerated by patients at low dosages. Further studies are also necessary to increase the understanding of flavonoids action in aggressive PCa and in PCa cells resistant to therapies. Dosage in vivo and the length of time for which cells are exposed to flavonoids may make the difference. Cancer cells may be sensitive to flavonoids activity when treated in specific “windows of opportunity”, which we should identify and take into account for the optimization of their use as natural epigenetic modulators for the chemoprevention, and possibly the treatment, of PCa and other kinds of cancer.

## Figures and Tables

**Table 1:** Structure of the main flavonoid compounds. Based on their structural differences, flavonoids can be divided into flavones, flavonols, flavanones, isoflavonoids, flavanols, and anthocyanins. Flavonoids whose epigenetic action is detailed in the text are in bold.

	Chemical Structure	Bonds	Compounds
FLAVONES	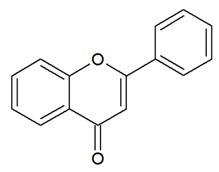	Double bond between C2-C3 and a ketone in C4 of the C ring.	Apigenin Luteolin Morin Tricin
FLAVONOLS	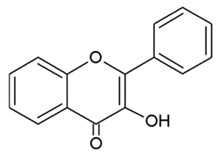	Hydroxylic group, a double bond between C2-C3 and a ketone in C ring.	Quercetin Myrecitin Fisetin Kaempferol
FLAVANONES	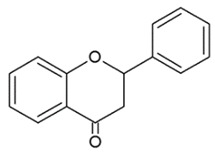	Lack the double bond between C2-C3 in C ring; only hydroxyl and methoxy groups as substituents.	Silibinin Naringenin Hesperdin
ISOFLAVONOIDS	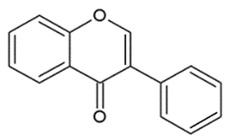	Large diversity of structure in the C ring. The B ring is attached at C3 rather C2 of the C ring.	Genistein DaidzeinGlycitein
FLAVANOLS OR CATECHINS	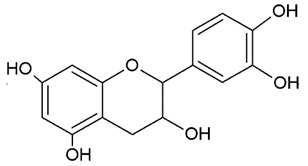	No double bond of C2-C3 in the hydroxyl group in position 3.	EGCG Epicatechin Epicatechin-3-gallate
ANTHOCYANINS	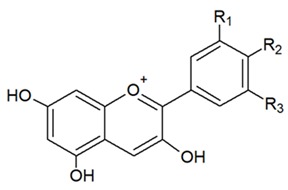	Flavylium cation binding hydroxyl groups and/or methoxy group(s) in R₁, R₂, and R₃ position.	Delphinidin Cyanidin PeonidinN

**Table 2:** Targets and cellular mechanism of flavonoids in prostate cancer (PCa). Flavonoids that exert anti-tumoral effect in PCa through epigenetic mechanisms are reported in the table. See text for details.

		Cell Lines/Animal Models/Clinical Trials	Molecular Target	Cellular Mechanism	Ref
*Flavones*	Apigenin	PC3-22Rv1 cells	↓ HDAC1, HDAC3 ↑ acetylation H3-H4	Cell cycle arrest apoptosis	[50]
		PC3 xenograft mice	↓ HDAC1, HDAC3	Tumor growth reduction	[50]
	Luteolin	PC3-LNCaP cells	↓ miR-301	Inhibition proliferation apoptosis	[121]
	+/- gefitinib	PC3 cells	↑ miR-603	Growth arrest Apoptosis	[124]
		PC3 cells	Binding to H4	Cell cycle arrest	[125]
	Morin + paclitaxel	DU145, PC3 cells DU145 xenograft mice	↓ miR-155 ↓ miR-155	Apoptosis Suppression of PCa progression	[131]
	Tricin + docetaxel	PC3 cells	↓ miR-21	Inhibition proliferation	[133]
*Flavonols*	Quercetin + hyperoside	PC3 cells	↓ miR-21	Apoptosis, cell cycle arrest and reduced invasive capacity	[139]
*Flavanones*	Silibinin	DU145, PC3 cells	↓ EZH2, ↑ DNMT ↓ HDAC1, HDAC2	Cell cycle arrest Apoptosis	[144]
*Isoflavonoids*	Genistein	LNCaP, LAPC-4 cells	↓ DNMT1, DNMT3b	Inhibition proliferation	[152]
		PC3, DU145 cells	↑ miR-34a, ↓ HOTAIR	Cell cycle arrest Apoptosis Inibition proliferation	[153]
*Catechins*	EGCG	PC3 cells	↓ DNMT	Cell growth inhibition	[165]
	EGCG	LNCaP cells	↓ HAT	Cell growth inhibition	[171]
	EGCG	LNCaP 22Rv1 cells 22Rv1 xenograft mice	↓ AR ↓ AR, miR-21, ↑ miR-330	Cell growth inhibition Suppression of PCa progression	[172]
	Polyphenon E	LNCaP, PC3 cells	↓ HDAC1, HDAC2, HDAC3, HDAC8 ↑ acetylation H3	Cell cycle arrest Apoptosis	[173]
	Polyphenon E	LNCaP cells	↓ DNMT1	Growth arrest	[174]
	Polyphenon E/EGCG	DUPRO, LNCaP cells Patients undergoing prostatectomy	↓ HDAC1, EZH2 ↓ HDAC1	Migration, invasion abrogation Suppression of PCa progression	[178]
*Anthocyanidins*	Delphinidin	LNCaP cell lines	↓ HDAC3	apoptosis	[181]

Histone deacetylase (HDAC), Enhancer of zeste homolog 2 (EZH2), histone acetyl transferase (HAT), DNA methyltransferase (DNMT), androgen receptor (AR), histone 3, 4 (H3, H4), HOX Transcript Antisense RNA (HOTAIR).
